# Significantly elevated foetal haemoglobin levels in individuals with glucose 6-phosphate dehydrogenase disease and/or sickle cell trait: a cross-sectional study in Cape Coast, Ghana

**DOI:** 10.1186/s12878-017-0088-6

**Published:** 2017-09-25

**Authors:** Patrick Adu, Essel K. M. Bashirudeen, Florence Haruna, Edward Morkporkpor Adela, Richard K. D. Ephraim

**Affiliations:** 1 Medical Laboratory Technology Department, School of Allied Health Sciences, University of Cape Coast, Cape Coast, Ghana; 2 Haematology unit, Cape Coast Teaching Hospital, Cape Coast, Ghana

**Keywords:** Haemoglobinopathy, Sickle cell trait, Glucose 6-phosphate dehydrogenase, Co-inheritance, Foetal haemoglobin

## Abstract

**Background:**

Previously published data have demonstrated that sickle red blood cells produce twice as much reactive oxygen species (ROS) suggesting that co-inheritance of sickle cell disease (SCD) and glucose 6-phosphate dehydrogenase (G6PD) enzymopathy could lead to more severe anaemia during sickling crises. Elevated foetal haemoglobin (Hb F) levels have been shown to have positive modulatory effects on sickling crises and disease outcomes. This study sought to assess how inheritance of G6PD enzymopathy affects the level of Hb F and haemoglobin concentration in adults in steady state.

**Methods:**

This cross-sectional study selected 100 out-patients (41 males and 59 females) visiting the University of Cape Coast hospital, between January, 2016 and May, 2016. Cellulose acetate electrophoresis (pH 8.2–8.6), methaemoglobin reductase test, modified Betke alkaline denaturation methods were used to investigate haemoglobin variants, qualitative G6PD status, and %Hb F levels in venous blood samples drawn from these participants. Data was analysed with GraphPad Prism 6 and SPSS and significance set at *p* < 0.05.

**Results:**

Forty one percent of the participants demonstrated qualitative G6PD enzymopathy whereas only 10% demonstrated Hb AS type (Sickle cell trait, SCT). 5% of the participants co-inherited SCT and G6PD enzymopathy. %Hb F levels in G6PD deficient males was significantly higher than in G6PD deficient females [(*p* = 0.0003, 2.696% (males) vs 1.975% (females)], although the %Hb F levels was comparable in non-G6PD deficient individuals. %Hb F levels were significantly elevated in males with SCT only (*p* < 0.05), or G6PD enzymopathy only (*p* < 0.0001), or SCT + G6PD enzymopathy (*p* < 0.0001) compared to males with none of these pathologies even though their respective haemoglobin levels were comparable. Male participants with G6PD enzymopathy + SCT co-inheritance had significantly elevated %Hb F when compared to their counterparts with only G6PD enzymopathy (*p* < 0.001). Male gender [(*p* = 0.001, OR: 6.912 (2.277–20.984)] partial defective G6PD enzyme [(p = 0.00, OR: 7.567E8 (8.443E7–6.782E9)] SCT [(*p* = 0.026, OR: 4.625 (1.196–17.881)] were factors associated with raised %Hb F levels ≥2.5.

**Conclusion:**

The inheritance of G6PD defect and/or SCT significantly elevate %Hb F levels in the steady state even though haemoglobin levels are not affected.

## Background

The normal physiologic functions of red blood cells (RBC) may be hampered by inherited haemoglobinopathies (e.g. sickle cell disease), red cell enzymopathy or red cell membrane abnormalities (e.g. G6PD deficiency). Although both G6PD enzymopathy and sickle cell are recessively inherited, whereas G6PD is sex-linked, sickle cell gene is autosomal inherited. SCD occurs due to the substitution of valine for glutamic acid in position 6 in the beta globin [1–3]. This then leads to the production of abnormal haemoglobin Hb S instead of normal haemoglobin Hb A [1, 4, 5]. Heterozygous inheritance leads to sickle cell trait (Hb AS) whereas homozygous inheritance leads to SCD with its consequent vaso-occlusive attacks, reactive oxygen species (ROS) generation, bacterial infections, priapism and chronic visceral complications often associated with ischemia in different organs [4, 6].

On the other hand, about 400 million people are estimated to be G6PD deficient worldwide [6–8]. In G6PD deficiency, terminally differentiated red cells become susceptible to oxidant stress-induced haemolytic anaemia due to absence of NADPH [5, 7, 9–11]. Agents causing oxidant stress in G6PD deficient individuals include fava beans and drugs such as aspirin, primaquine and quinine [4].

As these red cell pathologies are independently inherited, the potential for co-inheritance may be high especially in sub-Saharan Africa where either of these pathologies has been shown to provide protection against malaria infection [12, 13]. Previously published data have demonstrated that sickle red cells produce twice as much ROS such as hydrogen peroxide, superoxides and hydroxyl radicals, compared to normal cells [14]. This means that there is a possibility that SCD individuals with G6PD deficiency will have an increased tendency to frequent acute haemolytic crises as a consequence of ROS generated by sickled red cells. Studies investigating the potential modulatory effect of G6PD enzymopathy on severity of sickle cell anaemia has produced conflicting results. Whereas some studies found lower haemoglobin levels [15, 16], others found no effect on clinical manifestations, haemoglobin level and reticulocyte counts in such co-inherited cases [17]. Besides, in Ghana where co-inheritance of these two red cell pathologies are common [18], there is paucity of such data to inform clinical decisions. Moreover, the endemicity of malaria means that drugs such as quinine, primaquine, and amodiaquine that predispose G6PD deficient to oxidant stress may be routinely prescribed to malaria patients. This necessitates that factors that has the potential to modulate disease pathologies in co-inherited G6PD deficient and SCD ought to be explored. As foetal haemoglobin (Hb F) levels have been demonstrated to positively modulate SCD pathogenesis, we investigated the impact of the inheritance of G6PD enzymopathy and/or SCT on the %Hb F as well as haemoglobin levels in the peripheral blood of these patients in steady state.

## Methods

### Study design/study site

This cross-sectional study was conducted at the University of Cape Coast hospital in the Central region of Ghana from January to May 2016. The hospital has a bedding capacity of 65, and has an average yearly out-patient department (OPD) attendance and admissions of 61,509 and 2608 respectively. The hospital has OPD, accident and emergency (A & E) unit, Surgical ward, Medical ward, laboratory department, radiography unit and physiotherapy unit.

### Participants

A simple convenience sampling technique was used to recruit 100 participants (41 males and 59 females) aged 15–84 years. The rationale for the study was explained to all clients attending the OPD unit of the hospital during the period of the research. Only clients who gave written informed consent were consecutively recruited for the study. Individuals who had been transfused within three months to the period of the research, or pregnant or those taking medications known to cause haemolysis in G6PD deficient individuals [19] were excluded. Only consenting participants who has no history of any chronic disease were recruited for the study.

### Sample collection

4mls of whole blood was taken from each participant into an ethylenediaminetetraacetic acid (EDTA) anticoagulated tubes following standard protocols. The blood was used for G6PD screening, haemoglobin concentration, foetal haemoglobin estimation and haemoglobin electrophoresis.

### G6PD screening assay

G6PD screening was undertaken using the methaemoglobin reduction assay as described by Cheesbrough [20]. G6PD status was described as full defect (FD), partial defect (PD) or no defect (ND).

### Haemoglobin estimation

The haemoglobin levels of each participant was estimated using the URIT-12 haemoglobin meter (URIT Medical Electronic Co. Ltd., Guangxi, China) following manufacturer’s protocol [21].

### Foetal haemoglobin estimation

The level of foetal haemoglobin in each participant was estimated using the modified Betke alkali denaturation method [22, 23].

### Haemoglobin electrophoresis

Haemoglobin variants in the participants were determined using the cellulose acetate electrophoresis (pH 8.2–8.6) in accordance with previously published protocols [24]. Haemoglobin type was described as SCT (AS), or NEG (A).

### Statistical analysis

The data was inputted into Microsoft Office Excel 2007 and grouped into various categories and later analyzed using the GraphPad Prism 6 (GraphPad Software Inc., USA). Descriptive analysis was performed and results expressed as numbers and percentages. Data was analysed for normality using D’Agostino-Pearson test. Comparison between two groups were undertaken using two-tailed unpaired t-test (if passed normality test) or Mann–Whitney test (if it did not pass normality test). Multiple comparisons were undertaken either with One-Way ANOVA with Dunn’s post-testing (those that passed normality testing) or Kruskal-Wallis test with Tukey’s post-testing (those not passing normality testing). The relationship between parameters were explored using Pearson correlation coefficients. However, SPSS version 22 (IBM, USA) was employed to interrogate factors associated with raised %Hb F levels ≥2.5 through logistic regression analysis. *P* value less than 0.05 was considered statistically significant.

## Results

The G6PD status and haemoglobin variants of the participants were stratified per gender (Table 1). The mean age of the male and female participants was similar (*p* = 0.77; 33.5 years vs 34.5 years). Overall, 41% of the study participants demonstrated G6PD defect (31% full defect and 10% partial defect). Whereas 58.5% of the male participants showed G6PD full defect, only 11.9% of the female participants demonstrated G6PD full defect. Additionally, 10% of the study participants also had the SCT (Hb AS type).

**Table 1:** Qualitative red cell G6PD enzyme test stratified by gender

Parameter	Male (%) *N* = 41	Female (%) *N* = 59	Total (%)
Mean age (yrs)	33.50 ± 1.87	34.4 ± 2.43	
G6PD status			
G6PD FD	24 (58.50)	7 (11.90)	31 (31%)
G6PD PD	0 (0.00)	10 (16.90)	10 (10%)
G6PD ND	17 (41.50)	42 (71.20)	59 (59%)
Haemoglobin type			
Hb AS	6 (14.60)	4 (6.80)	10 (10%)
Hb A	35 (85.4.0)	55 (93.20)	90 (90%)

*G6PD* Glucose-6-phosphate Dehydrogenase, *FD* full defect, *PD* partial defect, *ND* no defect

The prevalence of co-inheritance of G6PD enzymopathy and SCT among the study participants is presented in Table 2. Overall, 5% (4 males vs 1 female) of the study participants co-inherited G6PD enzymopathy and SCT. Whereas 5% (2 males vs 3 females) of the study participants inherited SCT alone, 36% inherited G6PD enzymopathy alone (27 full defect vs 9% partial defect).

**Table 2:** G6PD Status + SCT co-inheritance of participants

G6PD status + SCT co-inheritance (*N* = 100)	Male (%)	Female (%)
G6PD ND + NEG	15 (15)	39 (39)
SCT + G6PD PD	0 (0)	1 (1)
SCT + G6PD FD	4 (4)	0 (0)
SCT + G6PD ND	2 (2)	3 (3)
G6PD FD + NEG	20 (20)	7 (7)
G6PD PD + NEG	0 (0)	9 (9)

*G6PD* Glucose-6-phosphate Dehydrogenase, *SCT* sickle cell trait, *FD* full defect, *PD* partial defect, *ND* no defect, *NEG* negative

**Fig. 1:**
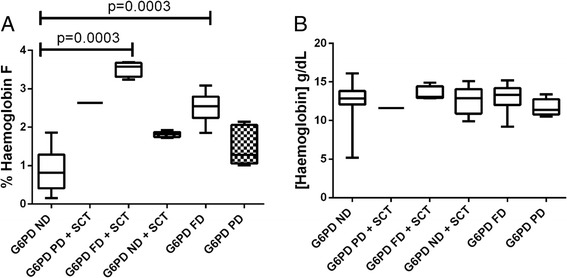
%Hb F (**a**) and haemoglobin (**b**) levels in participants stratified as per their qualitative G6PD and/or SCT status. [G6PD = Glucose-6-phosphate Dehydrogenase, SCT = Sickle Cell Trait, FD = Full defect, PD = Partial defect, ND = No defect]

Figure 1 shows the %Hb F and haemoglobin levels in participants stratified by their qualitative G6PD and/or SCT status. Participants who demonstrated qualitative G6PD full defect showed significantly higher levels of %Hb F than those with partial defect or normal qualitative G6PD activity [Fig. 1a, p=0.0003; G6PD ND (0.8726%) vs G6PD FD (3.523%); *p* = 0.0003; G6PD ND (0.8726%) vs G6PD FD + SCT (2.514%)]. However, although inheritance of SCT increased the % Hb F levels compared to those with neither G6PD enzymopathy nor SCT, the increment was not significant (*p* > 0.05). Haemoglobin levels did not significantly differ among the participants irrespective of their G6PD status and/or haemoglobin type (*p* > 0.05, Fig. 1b).

**Fig. 2:**
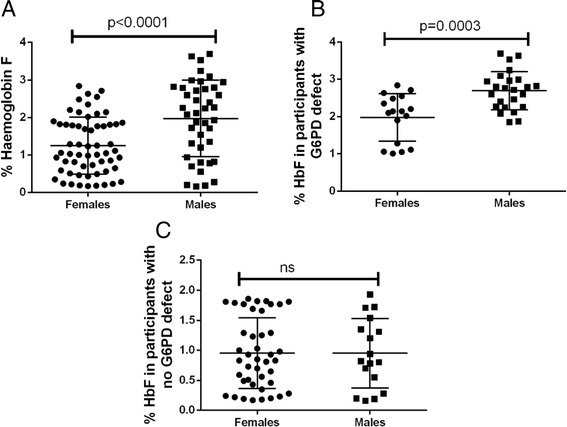
% Hemoglobin F and G6PD characteristics among study participants stratified by gender. **a** %Haemoglobin F comparison of all participants based on gender; **b** %Haemoglobin F of participants with G6PD defect stratified by gender; **c** %Haemoglobin F of participants with no G6PD defect stratified by gender

Figure 2 shows the % haemoglobin F among study participants stratified by gender. Overall, male participants had significantly higher %Hb F compared to female participants [Fig. 2a, *p*<0.0001; 1.247% (females) vs 1.973% (males)]. When the data was stratified based on the inheritance of G6PD enzymopathy, the significantly increased %Hb F levels was found only between males and females having G6PD enzymopathy [Fig. 2b, *p*=0.0003; 1.975% (females) vs 2.696% (males)]. Male and female participants with normal G6PD enzyme activity had comparable %Hb F levels (*p* = ns, Fig. 2c; 0.9524% (females) vs 0.9518% (males)].

**Fig. 3:**
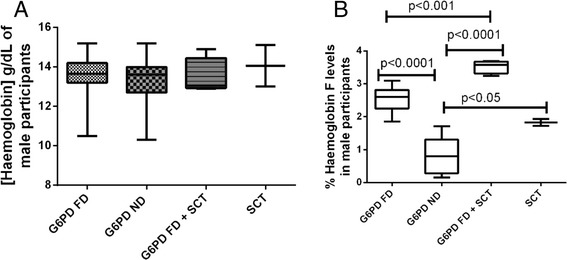
G6PD enzymopathy and/or SCT on %HbF levels in male participants. **a** compares the haemoglobin levels of male participants; **b** compares the %Hb F levels in male participants

Since the male participants had significantly elevated %Hb F compared to the females, we evaluated the impact of G6PD enzymopathy and/or SCT on %Hb F levels in male participants (Fig. 3). The haemoglobin levels did not significantly differ among the groups (Fig. 3a). However, the inheritance of G6PD enzymopathy and/or SCT was significantly associated with increased %Hb F levels in the male participants (Fig. 3b).

The study also explored the relationship between %Hb F, Hb and age of participants through Pearson correlation coefficients (Table 3). Age and %Hb F levels were inversely, but non-significantly correlated to each other (*r* = −0.022; *p* = 0.830). However, age and Hb levels as well as Hb and %Hb F levels were positively but non-significantly associated with each other.

**Table 3:** Correlations between age, Hb and %Hb F levels

Parameter		Age	Hb	%HbF
Age	r	1	0.143	−0.022
	*P*-value		0.156	0.830
Hb	r		1	0.094
	*P*-value			0.353
%HbF	r			1

r: Pearson correlation coefficient

Factors associated with raised %Hb F levels ≥2.5% were predicted using binary logistic regression analysis (Table 4). Male gender [*p* = 0.001, OR: 6.912, 95% CI (2.277–20.984)], partial defective G6PD enzyme [p = 0.00, OR: 7.567E8, 95% CI (8.443E7–6.782E9)] and haemoglobin variant AS [*p* = 0.026, OR: 4.625, 95% CI (1.196–17.881)] were all significantly associated with raised %Hb F levels ≥2.5.

**Table 4:** Factors associated with %Hb F levels ≥2.5

Parameter	OR (95% CI)	*P*-value
Age group		
< 20	0.958 (0.160–5.746)	0.963
20–29	1.420 (0.447–4.506)	0.552
30–09	0.548 (0.121–2.471)	0.433
≥ 40	Reference	–
Sex		
Male	6.912 (2.277–20.984)	**0.001**
Female	Reference	–
Haemoglobin (g/dL)		
< 12	0.610 (0.185–2.014)	0.417
≥ 12	Reference	–
G6PD status		
Normal	Reference	–
PD	7.567E8 (8.443E7–6.782E9)	**< 0.001**
FD	1.238 (1.238E10)	–
Haemoglobin variant		
A	Reference	–
AS	4.625 (1.196–17.881)	**0.026**

## Discussion

We sought to investigate the impact of the inheritance of G6PD enzymopathy and/or SCT on the %Hb F as well as haemoglobin levels in the peripheral blood of these patients in steady state. Our findings showed a 5% co-inheritance of SCT and G6PD enzymopathy and significantly elevated %Hb F levels in individuals with G6PD enzymopathy compared to either individual with SCT or normal G6PD enzyme activity. This suggests that Hb F levels may modulate the severity of G6PD enzymopathy in conditions of oxidant stress.

In the present study, a 41.0% prevalence of G6PD deficiency was recorded among the study population which is higher than the suggested Ghana’s 15–26% prevalence by the World Health Organization [25] or the estimated 1.2–30.7% prevalence in Africa [26]. This results is also at variance with a previous cross-sectional study undertaken in the Brong-Ahafo region of Ghana which recorded 32% G6PD enzymopathy prevalence among blood donors [27]. Many other studies in the sub-region and beyond have recorded lower prevalence in the past. For example, in a study in Iran, Nabavizadeh and Anushiravani reported a 14.17% prevalence of G6PD enzymopathy among 261 blood donors [28]. Also Omisakin et al., reported a 25.5% G6PD enzymopathy prevalence among blood donors in Nigeria [29]. These differences may be due to the different geographic locations as well as sampling frame employed in the various studies. Our study also found more males with G6PD enzymopathy compared to females [males (24.0% with full defect) vs females (females 7.0% with full defect and 10% with partial defect)]. This is not surprising considering that the genetic locus of the *G6PD* gene is on the X-chromosome and inherited in a recessive manner [30, 31].

The sickle-cell trait is known to be widespread, reaching its highest prevalence in parts of Africa as well as among people with origins in equatorial Africa. Previous studies have recorded high prevalence of SCT for countries such as Democratic Republic of Congo (23.3%) [32], Gabon (21.1%) [33], and Nigeria (19.68% - 45%) [34, 35], and Uganda (19.8%) [36]. It has been stated that in countries where the trait prevalence is above 20%, the SCD affects about 2% of the population. This study recorded a 10% SCT prevalence among the participants which is lower than the 19.5% SCT recorded among blood donors in Ghana [27]. However, this finding agrees with a previously published 11.3% SCT reported among blood donors in Ghana [37]. This study thus supports the estimated annual prevalence of SCD in Ghana with its associated high rates of morbidity and mortality [38].

Previous studies have indicated the co-existence of G6PD deficiency in patients with sickle cell disease [39, 40]. The prevalence of both diseases are highest in sub-Saharan Africa [41], and the Arabian Peninsula [42]. This study found a 5% prevalence of co-inherited SCT and G6PD enzymopathy which is comparable to the 7% prevalence of co-inherited G6PD enzymopathy and SCT recorded in a previous cross-sectional study in Ghana [27]. Another study in the sub-region by Egesie et al.*,* also recorded a 5.4% coinheritance of SCT and G6PD deficiency among blood donors in Nigeria [43]. This 5% co-inheritance reported herein is however at variance with reported co-inheritance prevalence reported elsewhere. For example Alabdulaali et al., reported SCT and G6PD co-inherited prevalence of 0.35% among blood donors in Riyadh, Saudi Arabia [44]. Another study that investigated the relationship between sickle cell disorders and G6PD deficiency in Central-Eastern India recorded a 0.61% prevalence of SCT and G6PD co-inheritance [45]. The differences in the prevalence reported in this study compared to the previous studies may be a function of the different selective pressures that exists in the different geographic locations where the studies were undertaken.

Studies investigating the potential modulatory effect of G6PD enzymopathy on severity of sickle cell anaemia has produced conflicting results. We report that, in the steady state, the haemoglobin levels of participants with SCT and/or G6PD enzymopathy did not significantly differ from participants with normal G6PD status and/or Hb A. Our study however found significantly raised %Hb F levels in males with G6PD enzymopathy compared to their female counterparts. Additionally, among the G6PD deficient males, the %Hb F levels were significantly elevated irrespective of the SCT status, when compared to the G6PD normal male counterparts. This observation is in agreement with a previous study in India that reported elevated %Hb F levels in G6PD deficient individuals [46]. Hb F has been shown to modulate the severity of haemoglobinopathies. In line with this, hydroxyurea is a pharmaceutical product used clinically to increase Hb F levels. Therefore, we propose that even though the haemoglobin levels did not differ in steady state, it is plausible to suppose that in haemolytic episodes, the elevated %Hb F levels in these defective G6PD and/or SCT may modulate the severity by improving oxygen transport to tissues and organs in the body. It is interesting to note that males who co-inherited SCT and G6PD enzymopathy had significantly elevated %Hb F compared to males who inherited either SCT or G6PD enzymopathy alone. In evaluating the factors associated with increased %Hb F levels ≥2.5%, our study also found male gender, partial defective G6PD enzyme and SCT as predictive of elevated %Hb F levels. This is in agreement with a previous cross-sectional study in Saudi Arabia that also found gender and haemoglobin variants as being associated with increased %Hb F levels [47]. However, whereas that study found age to be associated with elevated %Hb F levels, this study did not. This is not surprising considering that the El-Hazmi et al. study sampled both adults and cord blood taken from day old babies whereas this study recruited only adults (15–84 years).

To our knowledge, this is the first study in the sub-region to clearly demonstrate G6PD enzymopathy to be associated with elevated %Hb F levels. As the %Hb F levels were significantly elevated in those with G6PD enzymopathy compared to those with SCT, it will be interesting to compare the %Hb F levels in those with sickle cell anaemia to those with G6PD defect as well as assess the impact that %Hb F levels on the pathogenesis of oxidant stress in individuals with G6PD enzymopathy. However, the impact of our study was limited by the sample size, and the G6PD screening assay employed which is not as sensitive as the fluorescence method [24]. Additionally, our study did not estimate the reticulocyte count in the participants as reticulocytosis could have confounded the interpretation of the methaemoglobin reductase test.

## Conclusion

In the steady state the inheritance of G6PD defect and/SCT significantly elevate %Hb F, but not haemoglobin levels in the peripheral blood. Male gender, SCT and G6PD partial defect are factors associated with elevated %Hb F ≥ 2.5. Therefore, in the management of individuals with G6PD defect and/or SCT, %Hb F levels should be monitored to inform clinical decisions. Further studies employing animal models should be undertaken to understand the modulating effect of %Hb F levels on G6PD defect and/or SCT.

## Abbreviations

EDTA: Ethylenediaminetetraacetic acid
G6PD FD: G6PD full defect
G6PD ND: G6PD no defect
G6PD PD: G6PD partial defect
G6PD: Glucose 6-phosphate dehydrogenase
Hb F: foetal haemoglobin
Hb: haemoglobin
ROS: reactive oxygen specie
SCT: Sickle cell trait

## References

[1] Lervolino LG, Baldin PEA, Picado SM, Calil KB, Viel AA, Campos LAF (2011). Prevalence of sickle cell disease and sickle cell trait in national neonatal screening studies. Rev Bras Hematol Hemoter.

[2] Madegowda C, Rao C (2013). The sickle cell anemia health problems: traditional and modern treatment practices among the Soliga tribes at BR Hills**,** South India. Antrocom Online J Anthropol.

[3] Mavanga NM, Boemer F, Seidel L, Nkebolo A, Malafu AG, Gerard C (2013). Blood groups, hemoglobin phenotypes and clinical disorders of consanguineous YANSI population.

[4] Simpore J, Ilboudo D, Damintoti K, Sawadogo L, Maria E, Binet S, Nitiema H, Ouedraogo P, Pignatelli S, Nikiema J-B (2007). Glucose-6-phosphate dehydrogenase deficiency and sickle cell disease in Burkina Faso. Pak J Biol Sci.

[5] Uzoegwu PN, Awah FM (2006). Prevalence of sickle Haemoglobin and glucose–6–phosphate dehydrogenase deficiency genes in the populations of north west and south west provinces**,** Cameroon. Anim Res Int.

[6] Hoffbrand AV, Moss PAH, Pettit JE, editors. Essential hematology, 5th edn. UK: Published by Blackwell Publishing Ltd; 2006.

[7] Carter N, Pamba A, Duparc S, Waitumbi JN (2011). Frequency of glucose-6-phosphate dehydrogenase deficiency in malaria patients from six African countries enrolled in two randomized anti-malarial clinical trials. Malar J.

[8] Turgeon ML (2012). Clinical hematology: theory and procedures.

[9] Beutler E, Duparc S, Group GPDW (2007). Glucose-6-phosphate dehydrogenase deficiency and antimalarial drug development. Am J Trop Med Hyg.

[10] Clark TG, Fry AE, Auburn S, Campino S, Diakite M, Green A, Richardson A, Teo YY, Small K, Wilson J (2009). Allelic heterogeneity of G6PD deficiency in West Africa and severe malaria susceptibility. Eur J Hum Genet.

[11] Monteiro WM, Franca GP, Melo GC, Queiroz A, Brito M, Peixoto HM, Oliveira MRF, Romero GA, Bassat Q, Lacerda MV (2014). Clinical complications of G6PD deficiency in Latin American and Caribbean populations: systematic review and implications for malaria elimination programmes. Malar J.

[12] Santana MS, Monteiro WM, Siqueira AM, Costa MF, Sampaio V, Lacerda MV, Alecrim MG (2013). Glucose-6-phosphate dehydrogenase deficient variants are associated with reduced susceptibility to malaria in the Brazilian Amazon. Trans R Soc Trop Med Hyg.

[13] Mehta A, Mason PJ, Vulliamy TJ (2000). Glucose-6-phosphate dehydrogenase deficiency. Baillieres Best Pract Res Clin Haematol.

[14] Ali MSM, HMA-b S (2014). glucose 6 phosphate dehydrogenase deficiency screen among males patients with sickle cell disorders in Sudan. Am J Res Commun.

[15] Nouraie M, Reading NS, Campbell A, Minniti CP, Rana SR, Luchtman-Jones L, Kato GJ, Gladwin MT, Castro OL, Prchal JT (2010). Association of G6PD with lower haemoglobin concentration but not increased haemolysis in patients with sickle cell anaemia. Br J Haematol.

[16] Benkerrou M, Alberti C, Couque N, Haouari Z, Ba A, Missud F, Boizeau P, Holvoet L, Ithier G, Elion J (2013). Impact of glucose-6-phosphate dehydrogenase deficiency on sickle cell anaemia expression in infancy and early childhood: a prospective study. Br J Haematol.

[17] Steinberg MH, West MS, Gallagher D, Mentzer W (1988). Effects of glucose-6-phosphate dehydrogenase deficiency upon sickle cell anemia. Blood.

[18] Adu P, Simpong DL, Takyi G, Ephraim RK (2016). Glucose-6-phosphate dehydrogenase deficiency and sickle cell trait among prospective blood donors: a cross-sectional study in Berekum, Ghana. Adv Hematol.

[19] Young DS, Pestaner LC, Gibberman V (1975). Effects of drugs on clinical laboratory tests. Clin Chem.

[20] Cheesbrough M (2006). District laboratory practice in tropical countries.

[21] Supplier GD (2009). URIT: URIT-12 Hemoglobin meter; easy 3-step operation.

[22] Jonxis J, Huisman T (1956). The detection and estimation of fetal hemoglobin by means of the alkali denaturation test. Blood.

[23] Molden D, Alexander N, Neeley W (1982). Fetal hemoglobin: optimum conditions for its estimation by alkali denaturation. Am J Clin Pathol.

[24] Barabara J, Bain IB, Michael A, Laffan S, Lewis M (2011). Dacie and Lewis practical Haematology.

[25] WHO Working Group WHO (1989). Glucose-6-phosphate dehydrogenase deficiency. Bull World Health Organ.

[26] Moradkhani K, Mekki C, Bahuau M, Te VLT, Holder M, Pissard S, Préhu C, Rose C, Wajcman H, Galactéros F (2012). Practical approach for characterization of glucose 6-phosphate dehydrogenase (G6PD) deficiency in countries with population ethnically heterogeneous: description of seven new G6PD mutants. Am J Hematol.

[27] Simpong DL, Adu P, Bashiru R, Morna MT, Yeboah FA, Akakpo K, Ephraim RK (2016). Assessment of iodine status among pregnant women in a rural community in ghana - a cross sectional study. Arch Public Health.

[28] Nabavizadeh SH, Anushiravani A (2007). The prevalence of G6PD deficiency in blood transfusion recipients. Hematology.

[29] Omisikan CT, Esan AJ, Ogunleye AA, Ojo-Bola O, Owoseni MF, Omoniyi DP. Glucose-6-phosphate dehydrogenase (G6pd) deficiency and sickle cell trait among blood donors in Nigeria. Am J Pub Health Res. 2014;2(2):51–5.

[30] Akanni EO, Oseni BSA, Agbona VO, Tijani BA, Tosan E, Fakunle EE, Mabayoje VO (2010). Glucose-6-phosphate dehydrogenase deficiency in blood donors and jaundiced neonates in Osogbo, Nigeria. J Med Lab Diagn.

[31] Minucci A, Moradkhani K, Hwang MJ, Zuppi C, Giardina B, Capoluongo E (2012). Glucose-6-phosphate dehydrogenase (G6PD) mutations database: review of the “old” and update of the new mutations. Blood Cell Mol Dis.

[32] Agasa B, Bosunga K, Opara A, Tshilumba K, Dupont E, Vertongen F, Cotton F, Gulbis B (2010). Prevalence of sickle cell disease in a northeastern region of the Democratic Republic of Congo: what impact on transfusion policy?. Transfus Med.

[33] Delicat-Loembet LM, Elguero E, Arnathau C, Durand P, Ollomo B, Ossari S, Mezui-me-ndong J, Mbang Mboro T, Becquart P, Nkoghe D (2014). Prevalence of the sickle cell trait in Gabon: a nationwide study. Infect Genet Evol.

[34] Jeremiah ZA (2006). Abnormal haemoglobin variants, ABO and Rh blood groups among student of African descent in Port Harcourt, Nigeria. Afr Health Sci.

[35] Oludare GO, Ogili MC. Knowledge, attitude and practice of premarital counseling for sickle cell disease among youth in Yaba, Nigeria. Afr J Reprod Health. 2013;17(4):175–82. 24558793

[36] Ndeezi G, Kiyaga C, Hernandez AG, Munube D, Howard TA, Ssewanyana I, Nsungwa J, Kiguli S, Ndugwa CM, Ware RE (2016). Burden of sickle cell trait and disease in the Uganda sickle surveillance study (US3): a cross-sectional study. Lancet Glob Health.

[37] Antwi-Baffour S, Asare RO, Adjei JK, Kyeremeh R, Adjei DN (2015). Prevalence of hemoglobin S trait among blood donors: a cross-sectional study. BMC Res Notes.

[38] Antwi-Boasiako C, Frimpong E, Ababio G, Dzudzor B, Ekem I, Gyan B, Sodzi-Tettey N, Antwi D (2015). Sickle cell disease: reappraisal of the role of foetal haemoglobin levels in the frequency of vaso-occlusive crisis. Ghana Med J..

[39] Al-Nood H (2011). Thalassaemia and glucose-6-phosphate dehydrogenase deficiency in sickle-cell disorder patients in Taiz, Yemen/Thalassémie et déficit en glucose-6-phosphate déshydrogénase chez des patients atteints de drépanocytose à Taïz (Yémen). East Mediterr Health J.

[40] Benkerrou M, Alberti C, Couque N, Haouari Z, Ba A, Missud F, Boizeau P, Holvoet L, Ithier G, Elion J (2013). Impact of glucose-6-phosphate dehydrogenase deficiency on sickle cell anaemia expression in infancy and early childhood: a prospective study. Br J Haematol.

[41] Allison AC (1954). The distribution of the sickle-cell trait in East Africa and elsewhere, and its apparent relationship to the incidence of subtertian malaria. Trans R Soc Trop Med Hyg.

[42] Al-Gazali L, Alwash R, Abdulrazzaq Y (2005). United Arab Emirates: communities and community genetics. Public Health Genomics.

[43] Egesie OJ, Egesie UG, Jatau ED, Isiguzoro I, Ntuhun DB. Prevalence of sickle cell trait and glucose 6 phosphate dehydrogenase deficiency among blood donors in a Nigerian tertiary hospital. Afr J Biomed Res. 2013;16:143–7.

[44] Alabdulaali MK, Alayed KM, Alshaikh AF, Almashhadani SA (2010). Prevalence of glucose-6-phosphate dehydrogenase deficiency and sickle cell trait among blood donors in Riyadh. Asian J Transfus Sci.

[45] Balgir RS (2006). Do tribal communities show an inverse relationship between sickle cell disorders and glucose-6-phosphate dehydrogenase deficiency in malaria endemic areas of central-eastern India?. Homo.

[46] Balgir RS (2008). Hematological profile of twenty-nine tribal compound cases of hemoglobinopathies and G-6-PD deficiency in rural Orissa. Indian J Med Sci.

[47] El Hazmi MA, Warsy AS, Addar MH, Babae Z (1994). Fetal haemoglobin level--effect of gender, age and haemoglobin disorders. Mol Cell Biochem.

